# Reasons for opposition to posthumous reproduction and prior consent: attitudes of Jewish men during the ongoing armed conflict

**DOI:** 10.1186/s13584-025-00703-6

**Published:** 2025-07-21

**Authors:** Bella Savitsky

**Affiliations:** Department of Nursing, School of Health Sciences, Ashkelon Academic College, Yitshak Ben Zvi 12, Ashkelon, Israel

**Keywords:** Posthumous sperm retrieval (PSR), Posthumous reproduction, Qualitative research

## Abstract

Following the outbreak of armed conflict in Israel on October 7, 2023, a new directive permits both the spouse and parents of a deceased individual to initiate sperm extraction without the need for a court order or prior consent. A previous study revealed that nearly 37% of men in relationships opposed PSR at their partner’s request, and half of the men whose parents are alive opposed PSR at their parents’ request. The current study aims to explore the reasons behind this opposition and to understand the underlying attitudes of those who object to PSR.

The study population consists of 600 Jewish men aged 18–49, with data collected between February and March 2024 through a questionnaire.

Main objections for PSR following parental or partner`s request centered on the perceived “unethical nature of planned orphanhood”. The level of religiosity was significantly associated with this theme: the proportion of men who cited this reason for opposing PSR following a partner’s request was 33.3% among secular men, 42.9% among traditional men, and 70.4% among religious men (*p* =.007).

The most common reason for opposing the collection of prior consent before army enlistment was the belief that “Eighteen-year-old conscripts are just children who don’t understand anything”; “The moment of enlistment is not the right time to ask such a complex question” and “It is unethical to invest in any process that would create planned orphanhood”.

The study emphasizes the need for prior consent and highlights the need for culturally sensitive and ethically informed PSR policies.

## Introduction

Posthumous sperm retrieval (PSR) is a medical procedure used to collect and preserve sperm from a deceased individual for future use. To obtain viable sperm, the procedure should be performed shortly after death, ideally within 24 h [1–3]. Following retrieval, sperm can be preserved for decades through cryopreservation, which involves freezing sperm at very low temperatures [4]. Posthumous sperm retrieval typically yields a limited number of viable spermatozoa. The retrieved sperm is used exclusively in intracytoplasmic sperm injection (ICSI) procedures, given the low quantity and variable quality of the sample [5].

Israel’s nationalized healthcare system aligns with the country’s pronatalist stance [6], making its policy on PSR more supportive than in other countries. For instance, countries like France, Germany, Norway, Sweden, and Slovenia have instituted total bans on PSR, regardless of whether prior consent was given [7]. In the United Kingdom, Canada, and the USA, prior consent is needed [7].

In Israel, the female partner of the deceased can initiate PSR without requiring court authorization. However, court approval is necessary before the sperm can be used for reproduction. Parents of the deceased require court approval both to initiate PSR and to use the sperm for reproductive purposes [8]. Parents seeking to create genetic grandchildren through posthumous sperm retrieval can only do so by involving a single woman who is interested in using the sperm of a deceased, non-anonymous man to conceive and become the child’s mother [9, 10]. The only restrictions are an objection from the surviving partner or the absence of one living biological parent [11].

On October 7, 2023, Hamas launched an unprecedented and coordinated assault on Israel, marking the deadliest day in the nation’s history. Approximately 6,000 militants breached the border from Gaza, infiltrating Israeli communities and military installations. The attack resulted in the deaths of 1,195 individuals, including 725 Israeli civilians, 71 foreign nationals, and 379 soldiers and security personnel. The assault also led to the abduction of around 250 individuals, including civilians and soldiers, who were taken into Gaza. In response, Israel initiated a large-scale military operation, leading to a prolonged conflict with significant casualties on both sides. As of June 7, 2025, the Israel Defense Forces (IDF) reported that 891 soldiers had been killed since the beginning of the conflict [12].

Following the outbreak of armed conflict on October 7, 2023, and the resulting high number of male military casualties, a new directive was introduced, allowing both the spouse and parents of a deceased combatant to initiate sperm extraction immediately, without the need for a court order. Between October 7, 2023, and June 7, 2025, family members of IDF soldiers submitted 236 applications for postmortem sperm retrieval. Of these, 229 procedures were successful and seven were unsuccessful due to time constraints following death. The majority of requests (77%) were submitted by the soldiers’ parents. The overall success rate was 97%, based on 229 successful retrievals out of 236 attempts. Additionally, similar requests were submitted by the families of 19 civilian citizens [13].

There is a prevalent assumption that every man desires genetic continuity [13, 14], leading to the belief that PSR is universally sought. However, it is important to test this assumption in the context of post-mortem genetic continuity. Our previous study revealed that a significant proportion of men do not support PSR, regardless of whether the request comes from a partner or parents. The current study aims to explore the reasons behind this opposition and to understand the underlying attitudes of those who object to PSR.

## Objective

The study aims to quantify the prevalence of different perspectives within this population and to gain a deeper understanding of the reasons behind these attitudes, particularly in the context of cultural, ethical, and emotional factors.

## Methods

### Study design

This study employed a mixed-methods approach to examine men’s attitudes toward posthumous sperm retrieval (PSR). The quantitative component involved a survey of 600 men, with data collected during February–March 2024 using a structured questionnaire that included participants’ demographics, their involvement in the ongoing armed conflict, and their attitudes toward PSR.

Recruitment was conducted through multiple channels. Approximately 20% of participants were recruited via closed Facebook groups for men, where a link to an anonymous Google Forms questionnaire was shared. The remaining participants were reached following media exposure - interviews on radio and television, as well as announcements published in newspapers. At the start of the questionnaire, participants were provided with a brief explanation of the study’s purpose. The first item asked respondents to confirm their consent to participate; only those who agreed were granted access to the full questionnaire.

### Study variables on demographic characteristics

A range of demographic variables was collected to characterize the study sample. Relationship status was categorized as ‘’married or in a committed partnership’’ versus ‘’other’’. Parenting status referred to whether participants had biological children (yes/no). Country of birth was recorded as either Israel or another country. Education level was grouped into academic versus non-academic. Employment status included five categories: student, employed, serving in the regular army, in permanent military service, or other. Participants were also asked whether they had living parents (yes/no). The variable regarding same-sex relationships was derived from participants’ responses about the type of intimate relationships they were engaged in. Religiosity was assessed via self-identification, with participants asked to place themselves along a spectrum corresponding to four widely recognized cultural-religious identities: secular (nonobservant), traditional (partially observant), religious (fully observant), and ultra-orthodox (strictly observant). These groups reflect differing levels of religious observance and hold varying views on issues such as military service, gender roles, and the balance between democratic and religious law. For example, ultra-orthodox individuals typically advocate for a religious state and are mostly exempt from military service; religious individuals are observant but socially integrated and more likely to serve; traditional individuals represent a broad spectrum of partial observance; and secular individuals, while culturally Jewish, support secular governance and oppose religious control over civil matters.

In addition to demographic information, participants were asked about their involvement in the ongoing war with Hamas. Based on their responses, a categorical variable was constructed to indicate participation in combat roles, non-combat roles, or no participation.

### Study variables on attitudes toward PSR

Men were asked:

*“Would you agree that in the event of your unexpected death*,* your parents will use your sperm to bring a child/children into the world? (Your parents will become the grandparents of your child when born)”*.

*“Would you want your spouse to use your sperm in the event of your unexpected death to bring a child/children into the world?”*

*“Do you believe that during recruitment for regular military service*,* soldiers should be asked for instructions regarding posthumous sperm retrieval in the event of their death during their military service?”*

*“Do you believe that during reserve duty enlistment*,* soldiers should be asked for instructions regarding posthumous sperm retrieval in the event of their death during their service?”*

For these four questions, the following answers were suggested: “yes”, “no”, and “do not know.”

Those who answered “No” were asked to explain the reason for opposition.

### Analysis

The results were analyzed using a chi-square test for categorical variables, and statistical analysis was performed using IBM SPSS Statistics for Windows, Version 25 (IBM Corporation, Armonk, NY). For all analyses, a p-value of less than 0.05 was considered statistically significant.

For the qualitative component, free-text responses provided by men in response to open-ended questions were analyzed. These responses allowed participants to express their attitudes toward PSR in their own words, offering rich qualitative data. The analysis involved coding these responses and identifying recurring themes, providing deeper insights into the participants’ nuanced perspectives and emotional responses.

To ensure the reliability of the qualitative analysis, two independent researchers reviewed the free-text responses of a sub-sample of 50 participants. Each researcher independently read through the responses multiple times to familiarize themselves with the data. They then coded the texts, identifying key themes and patterns that emerged from the participants’ words. After completing their individual analyses, the researchers compared their findings to ensure consistency and resolve discrepancies. This collaborative process helped to strengthen the validity of the identified themes.

Ethical considerations: The study received approval from the Ethical Board of the Department of Nursing at the Ashkelon Academic College (#04.2024).

## Results

### Participants’ demographics

The study population included 600 men. Table 1 presents a detailed demographic profile of the study population, categorized by their level of involvement in the ongoing armed conflict (no participation, non-combat role, combat role). The average age of participants was 31.7 years (SD = 8.1). A minority (6.5%) reported being in same-sex relationships, and slightly more than half (52%) were married or in committed partnerships. Biological parenthood was reported by 39% of respondents. Regarding religious identification, 63.2% of participants described themselves as secular, while 20.6% identified as traditional, and 16.2% as religious. The vast majority (88%) were Israeli-born, with the remaining 12% being immigrants, primarily from the Former Soviet Union. A significantly higher proportion of traditional respondents was observed among combatants (27.2%) compared to non-combat participants (19.0%) and non-participants (16.2%) (*p* <.001). Among those who took part in the conflict, 4.8% were active-duty soldiers, 10.0% were in permanent military service, 13.9% were students in reserve service, and 66.5% were employed reservists.

**Table 1:** Characteristics of the study population

Demographic Characteristics		Participation in the current war with Hamas			*p*-value*	Total *n* = 600
		Participated in a combat role *n* = 209 35%	Participated in a non-combat role *n* = 124 21%	Did not participate *n* = 267 44%		
**Age (years)**,** mean (SD)**		30.5 (7.0)	30.0 (7.9)	33.4 (8.7)	< 0.001	31.7 (8.1)
**Family Status** **%**	Married/in a committed relationship	54.5	48.4	51.7	0.55	52.0
	Other	45.5	51.6	48.3		48.0
**Men in same-sex relationships %**		1.9	9.7	8.6	0.004	6.5
**Parenting for Biological Children %**	Yes	36.4	35.5	42.7	0.25	39.0
	No	63.6	64.5	57.3		61.0
**Country of birth %**	Israel	92.3	87.9	85.0	0.09	88.0
	Immigrant	7.7	12.1	15.0		12.0
**Level of religiosity %**	Secular	51.5	63.6	72.3	< 0.001	63.2
	Traditional	27.2	19.0	16.2		20.6
	Religious	21.4	17.4	11.5		16.2
**Education %**	Non-academic	38.3	31.5	35.6	0.45	35.7
	Academic	61.7	68.5	64.4		64.3
**Employment %**	Student	13.9	8.9	12.0	< 0.001	12.0
	Employed	66.5	55.6	76.0		68.5
	In regular army service	4.8	10.5	3.7		5.5
	In permanent army service	10.0	22.6	0.4		8.3
	Other	4.8	2.4	7.9		5.7
**Having living parents** **%**		84.2	87.1	83.5	0.66	84.5

*p-value for Age is based on ANOVA test; all other p-values are based on Chi-square tests comparing participation groups

### Opposition to PSR at parental request

**Fig. 1:**
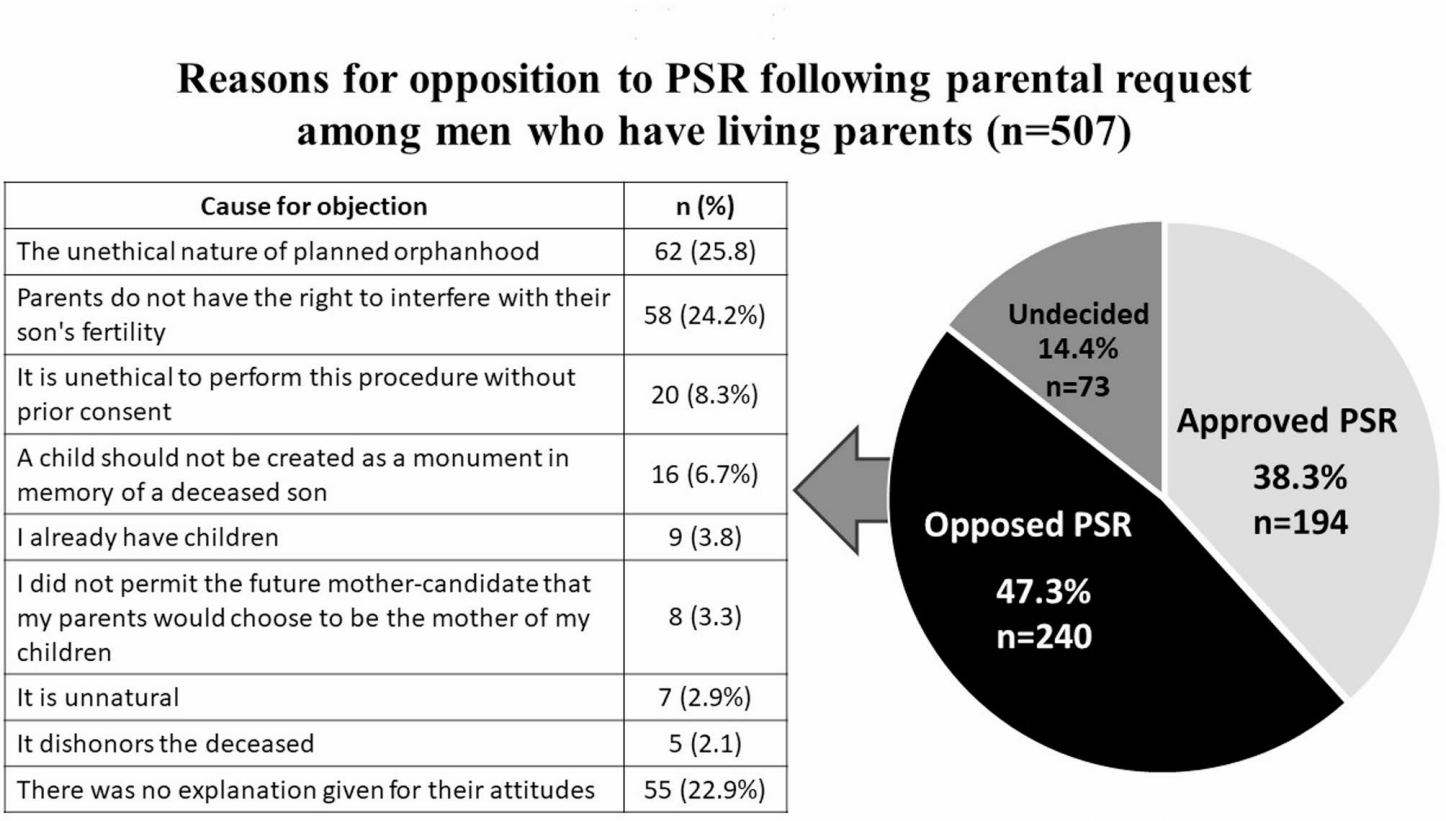
Reasons for opposition to PSR following parental request among men who have living parents (*n* = 507)

Most of the participants (*n* = 507) had both parents alive, and nearly half of these men (47.3%, *n* = 240) opposed PSR at their parents’ request, 38.3% (*n* = 194) approved of PSR following a parental request, and 14.4% (*n* = 73) were undecided. The reasons for opposition to PSR following parental request among men who have living parents are depicted in Fig. 1. The frequency of missing data (22.9% of 240 men who opposed PSR did not explain their attitudes) did not vary by demographic characteristics.

Opposition to PSR among 25.8% of respondents was based on the belief in the “unethical nature of planned orphanhood”. Those who cited this reason did not differ demographically from respondents who provided other reasons for their opposition. Examples of statements classified under this theme include: “There is no justification for bringing a child into the world already as an orphan, solely to fill the emotional void of a parent. This is a fundamentally selfish act”; “A child has the right to grow up with their father, and that right outweighs my personal desire for continuity”; “I have no right to bring a child into the world where I will not be present, relevant, or able to love and support them”; “It is a form of emotional harm to the child and to their future to bring them into a world where they must be told that their father died before they were born, and that they were conceived through the retrieval of his sperm”.

Another important reason for opposing PSR, as provided by the men, was that “Parents have no right to interfere with their son’s fertility”. This explanation was given by 24.2% of the men, and this attitude was similarly prevalent across different demographic groups.

A smaller proportion of men (8.3%) based their opposition on the belief in the “Unethical nature of requesting PSR without prior approval from their son for this procedure”.

The statement “A child is not a monument that is made in memory of the fallen son” was mentioned as a reason for opposition to PSR by 6.7%, and “I did not permit the future mother-candidate that my parents would choose to be the mother of my children”– by 3.3%. None of the men’s demographic characteristics were significantly associated with the themes.

### Opposition to PSR at the partner’s request

**Fig. 2:**
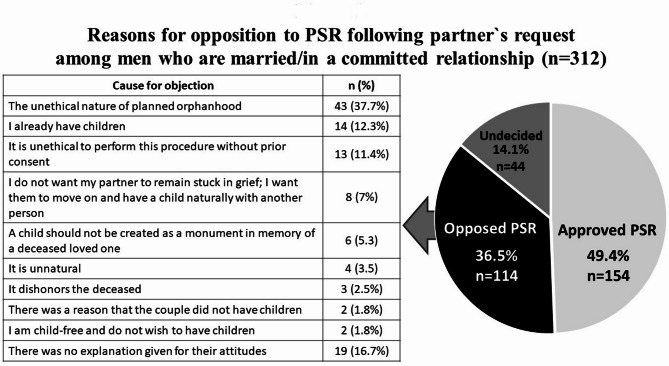
Reasons for opposition to PSR following partner`s request among men who are married/in a committed relationship (*n* = 312)

Half of the study population (*n* = 312) were married or in a committed relationship, where 36.5% of them (*n* = 114) opposed PSR at their partner’s request; 49.4% (*n* = 154) approved of PSR following a partner`s request, and 14.1% (*n* = 44) were undecided. The reasons for opposition to PSR following the partner’s request among men who are married/in a committed relationship are depicted in Fig. 2.

Of those 114 men who opposed, 16.7% did not explain their attitudes. Opposition to PSR among 37.7% was based on the “Unethical nature of planned orphanhood”. The level of religiosity was significantly associated with this theme: the proportion of men who cited this reason for opposing PSR following a partner’s request was 33.3% among secular men, 42.9% among traditional men, and 70.4% among religious men (*p* =.007).

Another frequent reason for opposing PSR, as provided by the men, was that “I already have children”. This explanation was given by 12.3% of the men. A smaller proportion of men based their attitude on the “Unethical nature of requesting PSR by the partner without consent for this procedure” (11.4%). 7% wrote, “I don’t want my partner to remain stuck in grief but to move on and have a child naturally with another man”. Finally, 5.3% based their opposition to PSR on the statement, “A child is not a monument that is made in memory of the fallen loved one”.

### Opposition to gathering prior consent before regular army service

Most men (71%, *n* = 423) believed that consent for PSR in the event of death during military service should be secured before enlistment, while 18.8% (*n* = 119) opposed this idea, and 9.7% (*n* = 58) were undecided.

Among those who opposed, 28.6% did not supply an explanation. The most frequent theme among those who opposed securing consent before enlistment (27.7%) was the belief that “Eighteen-year-old conscripts are just children who don’t understand anything”. Additionally, 15.1% stated, “The moment of enlistment is not the right time to ask such a complex question”. Another 9.2% argued that “It is unethical to invest in any process that would create planned orphanhood”, while an equal 9.2% opposed the idea of post-mortem sperm retrieval altogether. Furthermore, 5% expressed concern that “Asking about genetic continuity after death during recruitment could induce panic or fear among soldiers and negatively impact morale”. An additional 5% believed “There was no need to ask”, reasoning that “if a soldier has a partner, she should decide, and if he doesn’t, sperm should not be retrieved anyway”. None of the men’s demographic characteristics were significantly associated with the abovementioned themes.

### Opposition to gathering prior consent before every recruitment to reserve service

Most of the participants, 78.2% (*n* = 469), advocated for men to be consulted about PSR before every reserve service; 15.3% (*n* = 92) opposed this idea, and 6.5% (*n* = 39) were undecided. Among those who opposed, 39.1% did not explain why; 19.6% stated that “The moment of enlistment to reserves is not the right time to ask such a complex question”; 18.5% opposed the idea of post-mortem sperm retrieval; 7.6% claimed that " Asking about genetic continuity after death during recruitment into the reserves could induce panic or fear among soldiers and negatively impact morale " and 6.5% believed “There was no need to ask, reasoning that if a soldier has a partner, she should decide, and if he doesn’t, sperm should not be retrieved anyway”. None of the men’s demographic characteristics were significantly associated with the previously mentioned themes.

## Discussion

In the previous study [15] a multivariable model identified the level of religiosity as a factor associated with attitudes toward PSR performed at the request of parents or a partner. Traditional men were the most supportive, followed by secular men, while religious men were the least supportive.

Understanding the reasons for opposition to PSR is important for several reasons. First, it helps identify the ethical, legal, and emotional concerns that individuals or groups may have, enabling more informed and sensitive policy-making and clinical practices. Second, understanding the opposition can reveal cultural or religious beliefs that shape attitudes toward PSR, which is essential for developing guidelines that respect diverse perspectives. Lastly, exploring opposition can highlight potential areas of conflict or misunderstanding, offering opportunities for education and dialogue to address any misconceptions. Additionally, the proportion of those who oppose PSR is substantial: nearly 37% of men in relationships opposed PSR at their partner’s request, and half of the men whose parents are alive opposed PSR at their parents’ request. Thus, the views of this significant group should be studied and respected. The current study elucidates the reasons behind the opposition to PSR.

The primary theme of opposition among those who opposed PSR following a parental or partner’s request was the “Unethical nature of planned orphanhood”. This ethical problem was raised during every discussion on PSR in previous manuscripts or essays. One of the main criticisms of posthumous reproduction is the concern that it may not serve the child’s best interests. Critics argue that bringing a child into the world without a living parent could harm their well-being. They contend that the desire of an adult to have a child after their death should not outweigh the child’s fundamental right to have two living parents [16]. However, this perspective is challenged by the paradox of future individuals, known as the nonidentity problem, which argues that “actions cannot harm future individuals because they do not make them worse off than they would otherwise have been. Individuals can be harmed only if they are worse off than they otherwise would have been if a particular action had not occurred“ [17]. In addition, previous researchers have not shown any evidence of harm to children who are raised by a single parent [18], however, while extreme harm is unlikely, certain disadvantages, such as limited resources, increased stress, and potential emotional challenges, may still exist. Children raised in households with continuously married parents tend to fare better than those raised by single parents. This pattern is evident across important health and development indicators, such as physical health [19], mental well-being [20], and academic achievement [21]. These influences might be explained by lower socio-economic position of single parents, parenting stress, lack of social networks and support, and social stigma, which can influence maternal mental health and effective parenting [22]. In addition, the theme of worry about planned orphanhood is based on Israeli men’s investment in raising children and their desire to be partners in the process. In Israel, Jewish non-orthodox men are actively involved in childcare. A survey of 405 Jewish men found that the majority (62%) believed childcare responsibilities should be equally shared between parents [23]. Therefore, men’s concerns about their children’s well-being should not be overlooked. The positive scenario, in which bereaved parents find a single woman willing to become a mother and have a known father with the support of his family, fulfills multiple needs: the bereaved parents’ desire for a genetically related grandchild, the single woman’s need for extended family support, and the child’s need for genealogical continuity and a sense of belonging [24].

The proportion of men who objected to PSR following a partner’s request, citing the “The unethical nature of planned orphanhood”, was highest among religious men, followed by traditional, and was least frequent among secular participants. In religious Jewish contexts, the traditional family unit is highly valued. The intentional creation of a child who will be fatherless from birth may be seen as conflicting with religious ideals of family structure [25, 26]. Secular perspectives often prioritize individual reproductive rights and choices, potentially viewing posthumous reproduction as an extension of reproductive freedom [11]. In addition, single parenthood is more prevalent in secular Jewish households than among religious Jews [27].

While Judaism strongly values procreation, planned orphanhood complicates this mitzvah (commandment). Authorities debate whether posthumous reproduction fulfills the commandment, as the deceased parent cannot actively participate in raising the child [28]. Halakhic debates persist about whether a child born through posthumous reproduction is recognized as the deceased’s legal heir. Some rabbis grant inheritance rights, while others reject this, leaving the child in a precarious legal and religious position [29].

Another frequently mentioned reason for opposing PSR at the request of parents was the belief that “It is unethical to perform this procedure without prior consent”. Autonomy is one of four basic principles of medical ethics, together with beneficence, nonmaleficence, and justice. Autonomy means that **“**every human being of adult years and sound mind has a right to determine what shall be done with his own body.“ [30]. Collecting prior consent for sperm retrieval is crucial to respect the ethical principle of autonomy, specifically “procreational or reproductive autonomy,” as it ensures that an individual’s rights and personal decisions regarding their body and reproductive choices are fully honored [31]. In addition, Judaism has concerns about the procedures involved in PSR, such as the prohibition against desecrating a corpse or deriving benefit from it, especially if sperm was not preserved during the man’s lifetime [32, 33].

Another frequently raised objection to allowing PSR at the request of parents was the belief that “Parents do not have the right to interfere with their son’s fertility”. Parents’ desire for grandparenthood primarily reflects their wish to fulfill their child’s interest in genetic continuity. Additionally, it serves their own interests in preserving the family lineage and experiencing the role of a grandparent. Moreover, parents are not supposed to outlive their children; it’s against the natural order. They expect to watch their children grow into adulthood and enjoy the lives of their children and grandchildren. PSR following a parental request serves multiple purposes, with the desire to maintain a connection to their deceased son through his offspring being one of them [9, 34, 35]. This also ties into another theme expressed by men, which is the belief that “A child should not be created as a monument in memory of a deceased son”. Having a living child serve as a memorial to a deceased parent can create a complex emotional experience and a dual identity for the child, as they navigate their own life while also symbolically representing the life of the parent being honored [35]. Another issue that may influence the approval of PSR following the parental request is the quality of the relationship between the deceased son and his parents [16]. One of the study participants who opposed PSR following a parental request wrote, “I wouldn’t trust my parents to raise a cat, certainly not a child”. Although this is an extreme expression of doubt regarding parental ability, it clearly demonstrates that obtaining prior consent is crucial when parents wish to become grandparents after their son’s death.

Finally, regarding the opposition to PSR following a parental request, men were concerned by the idea that “I did not permit the future mother-candidate that my parents would choose to be the mother of my children”. Indeed, the previous study aimed, among other objectives, to identify the characteristics of a potential mother from the perspective of 212 Jewish unmarried male soldiers. It revealed that these soldiers had a specific vision of their desired qualities in a future mother [36]. Thus, giving prior consent, men should discuss this issue with their parents. Other reasons for opposing PSR following parents’ request were much less frequent (mentioned by less than 3% of the participants).

Regarding the opposition to PSR following the partner`s request, except for “The unethical nature of planned orphanhood” men were bothered by the “It is unethical to perform this procedure without prior consent”. As a deceased can’t express his wishes, respecting the deceased’s autonomy has long been established through honoring wills or expressed intentions [37]. This underlines the importance of preliminary written consent, which may help to prevent this problem [38].

The belief held by some men who oppose posthumous sperm retrieval (PSR) - that they “Do not want their partner to remain stuck in grief but rather move on and have a child naturally with another person” - reflects a compassionate and considerate perspective. It acknowledges the emotional complexities that come with loss and prioritizes their partner’s well-being by encouraging them to embrace new opportunities for love and family in the future. This attitude aligns with findings that demonstrate the correlation between new romantic relationships and improved psychological well-being, suggesting that moving forward can be a healthy and positive step in the healing process [39]. By advocating for their partner’s ability to find happiness again, these men demonstrate care and a selfless desire for their loved one to rebuild a fulfilling life.

The opposition to PSR, based on the belief that “A child should not be created as a monument in memory of a deceased loved one” which was raised in the context of parental requests, was also mentioned in relation to requests made by partners. While research on the well-being of children conceived through PSR is limited, some perspectives can be drawn from ethical analyses and discussions. If the child’s well-being is considered paramount, then it is essential to acknowledge the potential emotional hardship faced by a child conceived without any chance of knowing their father. The wish of an adult to bring a child into the world under such circumstances should not outweigh the child’s fundamental right to have two living parents, at least at the time of their conception [40]. Other reasons for opposing PSR following the request of a partner were much less frequent.

Regarding the importance of obtaining prior consent at the time of recruitment, almost 19% of participants opposed asking soldiers for consent before their regular service, and 15% opposed gathering consent before reserve service. For regular service, a common theme was the statement, “Eighteen-year-old recruits are just children who don’t understand anything”. This concern aligns with recent studies showing that the prefrontal cortex, crucial for higher cognitive functions and complex behavior, undergoes significant development during adolescence and does not fully mature until around the age of 25 [41]. An undeveloped brain is associated with higher vulnerability, which affects a higher risk of PTSD among soldiers. A study conducted among Vietnam veterans revealed that men who entered the war before the age of 25 were seven times more likely to develop PTSD compared to those who were older [42]. On the other hand, if eighteen-year-olds are considered mature enough to be recruited into the military, handle weapons, and risk their lives, it could be argued that they should also be asked about their consent to PSR in the event of their death.

The literature search did not provide evidence to support the concern that asking about PSR at recruitment might harm morale. However, the claim about “Inappropriate timing” is reasonable. The recruitment process focuses primarily on joining the military, and introducing topics like PSR, organ donation, or other death-related matters during routine medical discussions or general administrative procedures at this stage may seem premature and could potentially cause anxiety. This approach allows for a more thoughtful decision-making process without negatively impacting morale or causing undue concern during the sensitive recruitment phase. Rather than addressing this issue during the recruitment phase, it may be more suitable to introduce the topic later in a soldier’s career as part of routine medical checkups or general administrative procedures. This approach enables a more considered decision-making process without risking diminished morale or causing unnecessary distress during the sensitive recruitment phase.

In general, the percentage of men who believe that instructions regarding posthumous sperm retrieval should be collected before regular and reserve military service was very high. Therefore, it is not appropriate to ignore the desire to document these instructions while the men are still alive. This ensures that their reproductive autonomy and personal wishes are respected and that their consent is properly recorded for any potential future decisions.

## Conclusions

Several important conclusions are suggested from the findings of this study.

First, religious influence on attitudes. Traditional men are more supportive of PSR compared to secular and religious men, who are generally less supportive. Religious men were the most likely to oppose PSR due to concerns about “planned orphanhood,” followed by traditional and then secular men. This variation underscores the importance of considering religious and cultural contexts when addressing PSR.

Second, ethical concerns. A major reason for opposition to PSR is the perceived unethical nature of creating a child who will be fatherless from birth. This concern is rooted in the belief that a child should have both parents alive to ensure their well-being and uphold traditional family structures. The opposition is particularly strong among religious men, probably because of their emphasis on traditional family values and structure.

Third, parental rights vs. child’s wellbeing. Opposition to PSR at the request of parents is often based on the belief that parents should not interfere with their son’s reproductive choices. Additionally, concerns are raised about the child’s role as a living monument to the deceased parent and the potential emotional complexities involved.

Fourth, consent and procreational autonomy of the deceased. There is a significant concern about the ethicality of requesting PSR without the deceased’s prior consent. Respecting the autonomy of individuals, even after their death, highlights the necessity of obtaining explicit consent for PSR.

Lastly, the timing of consent. The study suggests that gathering consent for PSR during military recruitment may be problematic due to the age and psychological development of recruits. However, integrating the topic into routine discussions later in a soldier’s career could be more appropriate and less likely to affect morale negatively.

### Recommendations

Several key recommendations come out of this study.

Consent protocols: Implement robust protocols for obtaining prior consent for PSR among soldiers, who are considered a high-risk group, ensuring that individuals are fully informed and their autonomy is respected.

Tailored policy development: Develop policies and guidelines for PSR that consider individuals’ diverse religious and cultural beliefs. Engaging with various communities to understand their perspectives can lead to more respectful and inclusive practices.

Ethical considerations: Address the ethical concerns related to planned orphanhood in policy discussions. Ensuring that policies are sensitive to the potential emotional and developmental impacts on children born through PSR is crucial.

Parental involvement: Establish clear boundaries regarding parental involvement in decisions about PSR. It is essential to respect the deceased’s autonomy and ensure that any decisions made by parents align with the deceased’s previously expressed wishes.

Lastly, timing and integration: Introduce discussions about PSR in a manner sensitive to individuals’ developmental stage, particularly in high-stress environments like military service. Integrate these topics into routine medical or administrative procedures later in a soldier’s career to allow for informed decision-making without negatively impacting morale.

### Study limitations

The current study did not include ultra-orthodox men due to the limited internet usage among the ultra-orthodox community, which made it challenging to reach this group through online surveys [43].

## Data Availability

No datasets were generated or analysed during the current study.

## References

[1] Shefi S, Raviv G, Eisenberg ML, Weissenberg R, Jalalian L, Levron J et al. Posthumous sperm retrieval: analysis of time interval to harvest sperm. Hum Reprod [Internet]. 2006 [cited 2024 Apr 13];21:2890–3. Available from: https://pubmed.ncbi.nlm.nih.gov/16959804/10.1093/humrep/del232 16959804

[2] Gat I, Umanski A, Kaufman S, Kedem A, Avraham S, Youngster M et al. What can we learn about posthumous sperm retrieval after extra long-term follow-up? J Assist Reprod Genet [Internet]. 2022 [cited 2024 Apr 14];39:1661–5. Available from: https://pubmed.ncbi.nlm.nih.gov/35689734/10.1007/s10815-022-02535-8 PMC9365901 35689734

[3] Tumram NK, Bardale RV, Ambade VN. Sperm motility and viability extracted from penile tract of corpses: A preliminary study. Med Leg J [Internet]. 2016 [cited 2024 Apr 16];84:132–4. Available from: https://pubmed.ncbi.nlm.nih.gov/26883798/10.1177/0025817216633358 26883798

[4] Shahine L. What Is Sperm Freezing? [Internet]. 2022 [cited 2024 Aug 13]. Available from: https://www.forbes.com/health/family/what-is-sperm-freezing/

[5] Ahuja KK, Mamiso J, Emmerson G, Bowen-Simpkins P, Seaton A, Simons EG. Pregnancy following intracytoplasmic sperm injection treatment with dead husband’s spermatozoa: ethical and policy considerations. Hum Reprod [Internet]. 1997 [cited 2024 Aug 13];12:1360–3. Available from: https://pubmed.ncbi.nlm.nih.gov/9222030/10.1093/humrep/12.6.1360 9222030

[6] Editorial Board of the Yale International Relations Association. Be Fruitful And Multiply: The Role Of Israeli Pronatalist Policy. Yale Review of International Studies [Internet]. 2018 [cited 2024 Aug 13]; Available from: https://yris.yira.org/essays/be-fruitful-and-multiply-the-role-of-israeli-pronatalist-policy-in-the-pursuit-of-jewish-demographic-dominance-in-the-holy-land/

[7] Krebs JD. Any man can be a father, but should a dead man be a dad: an approach to the formal legalization of posthumous sperm retrieval and posthumous reproduction in the United States. Hofstra Law Rev [Internet]. 2018 [cited 2024 Apr 16];47:11. Available from: https://scholarlycommons.law.hofstra.edu/hlr/vol47/iss2/11

[8] Attorney General Israel. Posthumous Sperm Retrieval and Reproduction (Hebrew). Jerusalem; 2003 Oct. Report No.: 1.2202.

[9] Ram-Tiktin E, Gilbar R, Fruchter RB, Ben-Ami I, Friedler S, Shalom-Paz E. Expanding the use of posthumous assisted reproduction technique: should the deceased’s parents be allowed to use his sperm? Clin Ethics. 2019;14:18–25.

[10] Gilbar R, Ram-Tiktin E. It Takes a Village to Raise a Child: Solidarity in the Courts-Judicial Justification for Posthumous Use of Sperm by Bereaved Parents. Med Law Rev [Internet]. 2020 [cited 2024 Apr 23];28:317–41. Available from: https://pubmed.ncbi.nlm.nih.gov/31638702/10.1093/medlaw/fwz033 31638702

[11] Hashiloni-Dolev Y, Triger Z. The invention of the extended family of choice: the rise and fall (to date) of posthumous grandparenthood in Israel. New Genet Soc. 2020;39:250–70.

[12] Almadon I, Dvori N, Since. 2023, more soldiers have been killed than in any other year in the past 40 years. 2025 [cited 2025 Jun 8]; Available from: https://www.mako.co.il/news-military/2025_q1/Article-c9577c918a52491026.htm?utm_source=chatgpt.com

[13] Human Instinct. How Our Primevil Impulses Shape Our Modern Lives| NHBS Academic & Professional Books [Internet]. [cited 2025 May 21]. Available from: https://www.nhbs.com/human-instinct-book?srsltid=AfmBOorSRxTcG7mOR6davKByTzqkKhTA2EcZNPZ4W4GkDbf1uExWBrAd

[14] The Selfish Gene - Richard Dawkins. - Oxford University Press [Internet]. [cited 2025 May 21]. Available from: https://global.oup.com/academic/product/the-selfish-gene-9780198788607?cc=il&=en&.

[15] Savitsky B, Eldar-Geva T, Shvartsur R. Israeli men’s attitudes toward posthumous reproduction and prior consent amid ongoing armed conflict. Andrology [Internet]. 2025 [cited 2025 May 22];13. Available from: https://pubmed.ncbi.nlm.nih.gov/39287600/10.1111/andr.13757 39287600

[16] Simana S. Creating life after death: should posthumous reproduction be legally permissible without the deceased’s prior consent? J Law Biosci. 2018;5. 10.1093/jlb/lsy017 PMC6121062 30191068

[17] Cohen IG. Regulating reproduction: the problem with best interests. Minn Law Rev. 2012;96.

[18] Douglass A, Daniels K. Posthumous reproduction: A consideration of the medicthical, cultural, psychosocial and legal perspectives in the new Zealand context. Med Law Int. 2002;5. 10.1177/096853320200500402 14983884

[19] Weitoft GR, Hjern A, Haglund B, Rosén M. Mortality, severe morbidity, and injury in children living with single parents in Sweden: a population-based study. Lancet [Internet]. 2003 [cited 2024 Aug 14];361:289–95. Available from: https://pubmed.ncbi.nlm.nih.gov/12559862/10.1016/S0140-6736(03)12324-0 12559862

[20] Lipman EL, Boyle MH, Dooley MD, Offord DR. Child well-being in single-mother families. J Am Acad Child Adolesc Psychiatry [Internet]. 2002 [cited 2024 Aug 14];41:75–82. Available from: https://pubmed.ncbi.nlm.nih.gov/11800212/10.1097/00004583-200201000-00014 11800212

[21] de Lange M, Dronkers J, Wolbers MHJ. Single-parent family forms and children’s educational performance in a comparative perspective: effects of school’s share of single-parent families. School Effectiveness and School Improvement [Internet]. 2014 [cited 2024 Aug 14];25:329–50. Available from: https://www.tandfonline.com/doi/abs/10.1080/09243453.2013.809773

[22] Lut I, Woodman J, Armitage A, Ingram E, Harron K, Hardelid P, Protocol. Health outcomes, healthcare use and development in children born into or growing up in single-parent households: a systematic review study protocol. BMJ Open [Internet]. 2021 [cited 2024 Aug 14];11:43361. Available from: /pmc/articles/PMC7880085/10.1136/bmjopen-2020-043361 PMC7880085 33574152

[23] Anabi O. Masculinities in Israel– Current Attitudes [Internet]. The Heinrich Böll Foundation. 2021 [cited 2023 Jul 10]. Available from: https://il.boell.org/en/2021/10/22/masculinities-israel-current-state-of-affairs

[24] Bokek-Cohen Y, Ravitsky V. Parent-initiated posthumous-assisted reproduction revisited in light of the interest in genetic origins. J Med Ethics [Internet]. 2023 [cited 2024 Apr 23];49:357–60. Available from: https://pubmed.ncbi.nlm.nih.gov/35725302/10.1136/medethics-2022-108204 35725302

[25] Feder D, The Jewish Roots Of Family, Values. 2010 [cited 2024 Aug 18]; Available from: www.frc.org.

[26] Barlevy D, Werren S, Ravitsky V. Posthumous planning following fertility preservation: a study of adolescent cancer patients in Israel. New Genet Soc. 2020;39:271–87.

[27] Keysar A, Single-Parent Families' Participation In The Jewish Community. Joumal of Jewish Communal Servic. 1994.

[28] Hezi Margalit Y. Towards Establishing parenthood by agreement in Jewish law. Am Univ J Gend. 2018;26.

[29] Hezi Margalit Y. Using the sperm of a deceased person– between a dream come true and a nightmare. Rabbinical Authority [Internet]. 2024 [cited 2025 May 21]; Available from: https://journal.lawforum.org.il/margalit-family-law/

[30] Varkey B. Principles of Clinical Ethics and Their Application to Practice. Medical Principles and Practice. 2021. 10.1159/000509119 PMC7923912 32498071

[31] Johnston J, Zacharias RL. The future of reproductive autonomy. Hastings Cent Rep. 2017;47. 10.1002/hast.789 PMC9908087 29171894

[32] Post-Mortem Sperm Retrieval \, Halperin M. M.D. [Internet]. [cited 2025 May 21]. Available from: https://www.daat.ac.il/daat/kitveyet/assia_english/halperin1-1.htm

[33] Should Israel allow posthumous sperm donation?. - The Jerusalem Post [Internet]. [cited 2025 May 21]. Available from: https://www.jpost.com/judaism/article-714504

[34] Ram-Tiktin E, Gilbar R. Solidarity as a Theoretical Framework for Posthumous Assisted Reproduction and the Case of Bereaved Parents. Ethical Theory and Moral Practice [Internet]. 2019 [cited 2024 Apr 29];22:501–18. Available from: https://link.springer.com/article/10.1007/s10677-019-10012-y

[35] Morgan LW, Morgan HG. The legal and medical ethics of Post-Mortem sperm retrieval on behalf of grandparents. Ethics Post-Mortem Sperm Retr. 2020;33:67–98.

[36] Bokek-Cohen Y, Ravitsky V. Soldiers’ preferences regarding sperm preservation, posthumous reproduction, and attributes of a potential posthumous mother. Omega (United States). 2019;79:132–56. 10.1177/0030222817725179 28799832

[37] Cutler EKM. Ethical considerations on the creation of life after death: an exploration of the status of posthumous assisted reproduction [Internet]. Temple University; 2022 [cited 2023 Dec 23]. Available from: https://scholarshare.temple.edu/handle/20.500.12613/7771

[38] Kindregan CP. Dead Soldiers and Their Posthumously Conceived Children. Journal of Contemporary Health Law & Policy [Internet]. 2015 [cited 2023 Dec 23];31:74. Available from: https://scholarship.law.edu/jchlp/vol31/iss1/5

[39] Schneider DS, Sledge PA, Shuchter SR, Zisook S. Dating and remarriage over the first two years of widowhood. Ann Clin Psychiatry [Internet]. 1996 [cited 2024 Sep 9];8:51–7. Available from: https://pubmed.ncbi.nlm.nih.gov/8807029/10.3109/10401239609148802 8807029

[40] Landau R. Posthumous sperm retrieval for the purpose of later insemination or IVF in Israel: an ethical and psychosocial critique. Human Reproduction [Internet]. 2004 [cited 2024 Sep 9];19:1952–6. Available from: 10.1093/humrep/deh360 10.1093/humrep/deh360 15243009

[41] Arain M, Haque M, Johal L, Mathur P, Nel W, Rais A et al. Maturation of the adolescent brain. Neuropsychiatr Dis Treat [Internet]. 2013 [cited 2024 Aug 18];9:449. Available from: /pmc/articles/PMC3621648/10.2147/NDT.S39776 PMC3621648 23579318

[42] Dohrenwend BP, Turner JB, Turse NA, Lewis-Fernandez R, Yager TJ. War-Related posttraumatic stress disorder in black, hispanic, and majority white Vietnam veterans: the roles of exposure and vulnerability. J Trauma Stress. 2008;21:133–41. 18404630 10.1002/jts.20327 PMC2538409

[43] Malach G. Two Thirds of Ultra-Orthodox Are Online. The Israel Democracy Institute [Internet]. 2021. Available from: https://en.idi.org.il/articles/37838

